# Expression of cyclin D1 in clear cell sarcoma of kidney. Is it useful in differentiating it from its histological mimics?

**DOI:** 10.1186/s13000-019-0790-8

**Published:** 2019-02-08

**Authors:** Nasir Uddin, Khurram Minhas, Jamshid Abdul-Ghafar, Arsalan Ahmed, Zubair Ahmad

**Affiliations:** 1 Department of Pathology and Laboratory Medicine, Aga Khan University Hospital, Karachi, Pakistan; 2 Department of Pathology and Laboratory Medicine, French Medical Institute for Mothers and Children (FMIC), Kabul, Afghanistan

**Keywords:** Clear cell sarcoma of kidney, Cyclin D1, Ewing sarcoma, Mesoblastic nephroma, Wilms tumor, Neuroblastoma

## Abstract

**Background:**

Clear cell sarcoma of the kidney (CCSK) is a rare malignant pediatric renal neoplasm with a heterogeneous histological appearance which often results in misdiagnosis. There are no specific immunohistochemical markers which can help in differentiating CCSK from other pediatric renal neoplasms. Recently Cyclin D1 has been investigated as a possible marker in this regard. In this study, we aim to determine the usefulness of Cyclin D1 in differentiating between CCSK and other pediatric renal neoplasms and to compare our results with those of recently published studies.

**Methods:**

A total of 48 cases of CCSK, Wilms tumor (WT), renal rhabdoid tumor, mesoblastic nephroma, renal Ewing sarcoma and neuroblastoma were included in the study. All cases were stained with cyclin D1. Extent of Cyclin D1 staining was graded according to percentage of positive tumor cells as diffuse (> 70%), focal (5 to 70%), and negative (< 5%). Intensity of Cyclin D1 staining was graded as strong or 3+, moderate or 2+ and weak or 1 + .

**Results:**

Most or all cases of CCSK, neuroblastoma and renal Ewing sarcoma demonstrated diffuse and strong positivity for Cyclin D1. Most cases of Wilms tumor (epithelial component) also demonstrated diffuse and often strong positivity for Cyclin D1. In most cases of WT, blastemal component was negative.

**Conclusions:**

Cyclin D1 is a sensitive but not specific immunohistochemical marker for CCSK and many other pediatric renal malignant neoplasms as well as for neuroblastoma. Hence, careful examination of histological features is important in reaching an accurate diagnosis in CCSKs. However, Cyclin D1 is very helpful in distinguishing between blastema-rich WT and CCSK.

## Background

Clear cell sarcoma of kidney (CCSK) is an uncommon mesenchymal renal tumor of uncertain histogenesis which comprises approximately 3 to 4% of malignant pediatric renal neoplasms. It occurs in young children and mean age at diagnosis is approximately 36 months. It is more common in males (male to female ratio is 2:1), is centered in the renal medulla and is almost always unifocal. It is usually circumscribed but unencapsulated with a tan, soft cut surface and most tumors are quite large in size. On histological examination, classic CCSK is characterized by nests or cords of epithelioid cells with round to oval nuclei. Myxoid pools and thin and regularly branching fibrovascular septa separate the cords of tumor cells [1–6].

However, CCSK is very heterogeneous and diverse in histological appearance and a number of variant histological patterns may be seen. It has a propensity to metastasize to bone, and “bone metastasizing renal tumor of childhood” is one of its synonyms [7]. In the past, its prognosis was poor but since the addition of doxorubicin to chemotherapeutic protocols (previously comprising vincristine and dactinomycin) secondary to the results of the first three National Wilms Tumor Study (NWTS) trials, survival has increased from 20 to 70%. All patients with CCSK, regardless of stage, are now treated with doxorubicin. Brain metastases have now become more common than bone metastases [2, 5, 8, 9]. Approximately 10% of CCSKs demonstrate a rearranging chromosomal translocation i.e. t(10,17) (q22;p13) resulting in *YWHAE-FAM22- NUTM2B/E* gene fusion which is also seen in high grade endometrial stromal sarcoma. Recently, CCSK has also been shown to consistently demonstrate BCOR gene abnormalities including exon 15 internal tandem duplications and *BCOR-CCNB3* gene fusion which distinguish it from other pediatric renal tumors. Metastases can occur as late as ten years after initial diagnosis. Owing to the essential role of doxorubicin in the therapy of CCSK, it is imperative that pathologists identify it accurately. Failure to do so can prevent a child from getting optimal chemotherapy [10–18].

Owing to its marked histological heterogeneity, CCSK can be mimicked by a number of malignant pediatric renal and extrarenal neoplasms including blastema-rich Wilms Tumor (WT), mesoblastic nephroma, neuroblastoma etc. It is often difficult to diagnose CCSK from these tumors on morphology alone [3, 5]. Until recently, there were no specific immunohistochemical markers that reliably distinguished CCSK from other pediatric renal neoplasms [5]. Thus, it was sometimes difficult to diagnose CCSK accurately (in spite of the therapeutic and prognostic importance of an accurate diagnosis). Molecular tests for detecting *BCOR* gene abnormalities are not available especially in developing countries such as ours. Recently, a number of studies have suggested that immunohistochemical stain Cyclin D1 is useful in distinguishing CCSK from some pediatric renal neoplasms [3, 5, 19]. More importantly, recent studies suggest that BCOR immunohistochemistry appear to be highly sensitive and specific for the diagnosis of CCSK based on the recently identified BCOR gene abnormalities. BCOR immunohistochemistry appears to be more specific than Cyclin D1 [14, 15].

The aim of the present study was to determine the usefulness of Cyclin D1 in distinguishing CCSK from other pediatric renal neoplasms and to see whether our findings match those in other recently published studies.

## Methods

### Study cohort

The surgical pathology files of the Section of Histopathology, Department of Pathology and Laboratory Medicine, Aga Khan University Hospital and cases were submitted from French Medical Institute for Mothers and Children were searched for cases of CCSK, WT (nephroblastoma), renal rhabdoid tumor (RT), congenital mesoblastic nephroma, Ewing sarcoma of kidney as well as retroperitoneal neuroblastoma reported over a ten year period i.e. January 1, 2008 and December 31, 2017. A total of 48 cases were included in the study. These included 19 cases of CCSK, 9 cases of WT and 4 cases each of renal RT, Mesoblastic nephroma and Ewing sarcoma. In addition, 8 cases of neuroblastoma were also included. Although neuroblastomas are not renal tumors but are retroperitoneal like the former and occur in the same age group as pediatric renal tumors. Due to these features they need to be distinguished from pediatric renal tumor. Hence, they were included in the study.

### Immunohistochemistry

All cases were reviewed by the two principal authors (NU and ZA). A number of immunohistochemical markers including Vimentin (Flex Monoclonal Mouse Antivimentin, clone V9, ready to use, DAKO, Glostrup, Denmark), Antismooth muscle actin (Flex Monoclonal Mouse Antismooth muscle actin, clone 1A4, ready to use, DAKO, Glostrup, Denmark), S100 protein (Flex Polyclonal Mouse Anti-S100, ready to use, DAKO, Glostrup, Denmark), CD99 (Flex Monoclonal Mouse Anti human CD99, clone V9, clone 12E7,ready to use, DAKO, Glostrup, Denmark), WT1 (Flex Monoclonal Mouse Antihuman, Wilms Tumor 1 (WT1) protein, clone 6F-H2–7, ready to use, DAKO, Glostrup, Denmark), Neurofilament (Flex Monoclonal Mouse Antihuman neurofilament protein, clone 2F11, ready to use, DAKO, Glostrup, Denmark), CD56 (Flex Monoclonal Mouse Antihuman CD56, clone 1223C3, ready to use, DAKO, Glostrup, Denmark), Synaptophysin (Flex Monoclonal Mouse Antihuman synaptophysin, clone DAKO SYNAP, ready to use, DAKO, Glostrup, Denmark), Desmin (Flex Monoclonal Mouse Antihuman desmin, clone D33, ready to use, DAKO, Glostrup, Denmark), Cytokeratin AE1/AE3 (Flex Monoclonal Mouse Antihuman cytokeratin, clone AE1/AE3, ready to use, DAKO, Glostrup, Denmark), Epithelial membrane antigen (Flex Monoclonal Mouse Antihuman epithelial membrane antigen, clone E29, ready to use, DAKO, Glostrup, Denmark) were performed in different cases at the time of initial reporting and were reviewed by the authors. Cyclin D1 (Flex Monoclonal Rabbit Antihuman Cyclin D1, clone EP12, ready to use, DAKO, Glostrup, Denmark) was retrospectively performed in all 48 cases. The staining for Cyclin D1 was performed on DAKO automated immunostainer. The antibody was optimized using Ventana DAB detection kit (Ventana Medical Systems) and standard quality control procedures were performed. Cyclin D1 staining was performed following heat induced antigen retrieval using ER2 antigen retrieval buffer. Cases of mantle cell lymphoma were used as positive control. Negative controls were also used. For the purpose of study, extent of Cyclin D1 staining was graded according to percentage of positive tumor cells as diffuse (> 70%), focal (> 5 to 70%) or negative (< 5%); intensity of staining was graded as strong or 3+ (nuclear intensity similar to that of mantle cell lymphoma control), moderate or 2+ (definite nuclear staining weaker than 3+ but easily identifiable at × 40 magnification) and weak or 1+ (nuclear staining identifiable only at high power magnification). This scale for extent and grading was based on that used in two studies published in 2015 [3, 5]. All cyclin D1 stained slides were also reviewed by the two principal authors (NU and ZA). All data was analyzed in SPSS software version 19.

## Results

A total of 48 cases were included in the study. These included 19 cases of CCSK, 9 cases of WT and 4 cases each of renal RT, Mesoblastic nephroma and Ewing sarcoma. In addition, 8 cases of neuroblastoma were also included.

Patients with CCSK ranged in ages from 1 to 29 years with means and median age of 5.5 and 3 years respectively. Of the 19 patients, 13 (68.4%) were males while 6 (31.6%) were females. Tumor was located in left and right kidney in 8 cases each. In 3 cases, laterality was not known. Tumor size ranged from 8 cm to 14.5 cm with mean tumor size of 11 cm in greatest dimension**.**

**Fig. 1:**
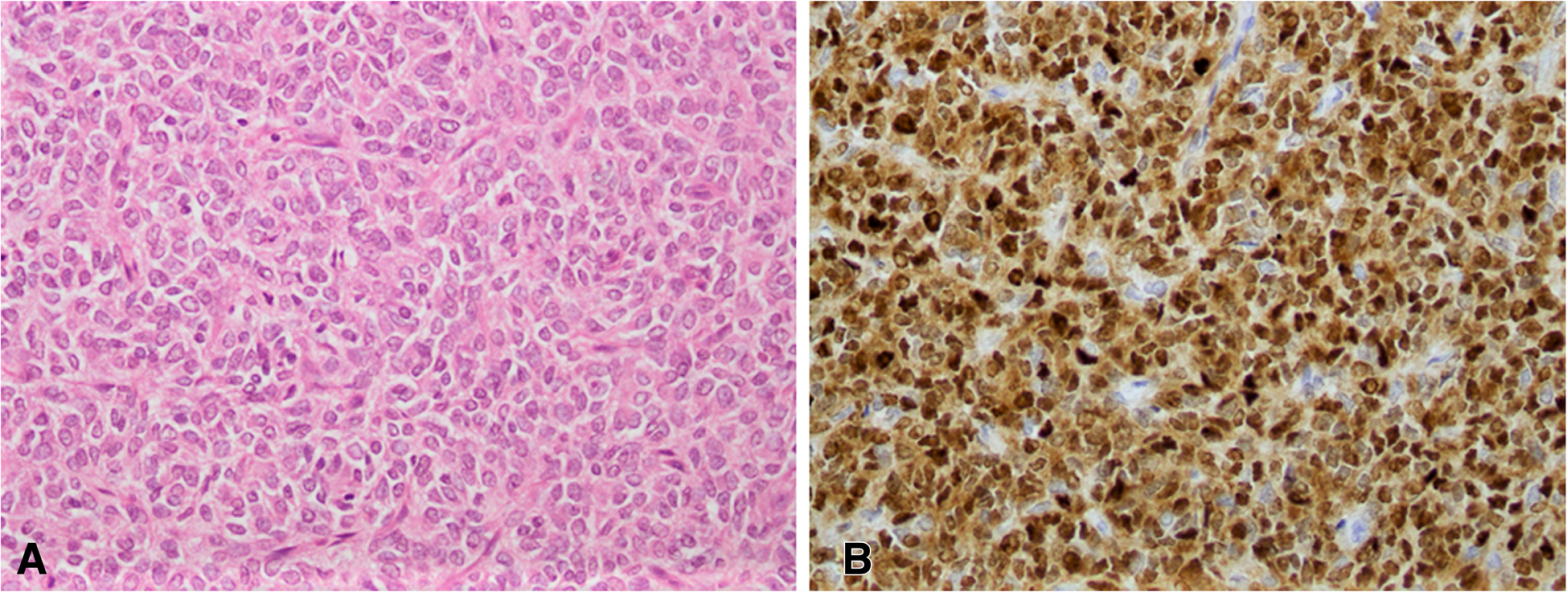
Classic clear cell sarcoma of kidney (**a**), showing diffuse strong Cyclin D1 positivity (**b**)

All 19 cases of CCSK demonstrated reactivity for Cyclin D1. In 13 cases (68.4%), intensity of staining was 3+, in 4 cases (21%), intensity was 2+, while in 2 cases (10.5%), it was 1+. Extent of staining ranged from 5 to 90% with average extent of staining being 54.5% (Fig. 1A,B). Extent of staining was 60% in 11 cases (57.9%), 50% in 3 cases (15.8%) and 90% in 1 case (5.3%). The clinical details as well as details of Cyclin D1 staining intensity and extent as well as details of other immunohistochemical markers are shown in Table 1.

**Table 1:** Clinical details and results of immunohistochemical staining with Cyclin D1 and other IHC markers in clear cell sarcoma of kidney (*n* = 19)

Age (years)	Sex	Site	Size (cm)	Cyclin D1 staining Intensity	Cyclin D1 staining proportion	ASMA	Desmin	S100	CKAE1	Vimentin	EMA	Bcl2	WT1	CD99
2	M	R	NK	2	60	-ve	-ve	-ve	-ve	+ve	NP	NP	NP	NP
29.5	M	R	12	1	5	-ve	NP	NP	-ve	+ve	-ve	NP	NP	NP
4	M	L	14.5	2	50	NP	NP	NP	-ve	+ve	NP	NP	NP	NP
4	M	L	12.5	3	60	NP	–ve	NP	-ve	+ve	NP	NP	NP	NP
2	M	R	9	3	50	-ve	-ve	-ve	-ve	+ve	NP	+ve	NP	NP
3	F	NK	NK	3	60	NP	-ve	NP	-ve	+ve	-ve	+ve	-ve	-ve
2	M	L	13.5	3	60	NP	NP	NP	-ve	NP	NP	+ve	-ve	-ve
1	M	R	7.5	3	60	NP	NP	NP	NP	+ve	-ve	+ve	-ve	-ve
1	M	L	8	1	5	-ve	-ve	-ve	-ve	+ve	NP	NP	-ve	NP
5	M	L	13	3	50	NP	NP	NP	-ve	+ve	-ve	NP	-ve	+ve
3	M	L	10	3	60	NP	NP	NP	-ve	+ve	-ve	+ve	-ve	NP
2.5	M	L	15	3	60	NP	NP	NP	-ve	+ve	-ve	+ve	-ve	NP
25	M	L	12	3	60	NP	NP	NP	-ve	+ve	-ve	NP	NP	-ve
5	F	R	9.3	3	60	NP	-ve	NP	-ve	+ve	NP	NP	NP	NP
1	F	R	7.5	2	20	NP	-ve	NP	NP	NP	NP	NP	-ve	+ve
5	F	R	9.9	3	60	NP	NP	NP	-ve	+ve	-ve	+ve	NP	NP
3	F	NK	17	3	60	NP	NP	-ve	-ve	+ve	NP	NP	NP	NP
1	F	R	7.5	2	15	NP	-ve	NP	NP	NP	NP	NP	-ve	Patchy +ve
1	M	NK	10	3	90	NP	NP	NP	-ve	+ve	NP	NP	-ve	+ve

IHC: immunohistochemical; ASMA: Anti-smooth muscle actin; M: Male; F: female; R: Right; L: Left; NK: not known; NP: Not performed

Of the 9 patients with WT, 6 (66.7%) were females, while 3 (33.3%) were males. Ages ranged from 2.5 to 7 years. Mean and median ages were 4.5 and 4 years respectively. In 6 patients (66.7%), tumor was located in the left kidney while in 3 (33.3%), tumor was located in the right kidney. Tumor size was available in 8 out 9 cases and ranged from 7.5 to 17.4 cm in largest dimension with mean tumor size of 11.5 cm.

**Fig. 2:**
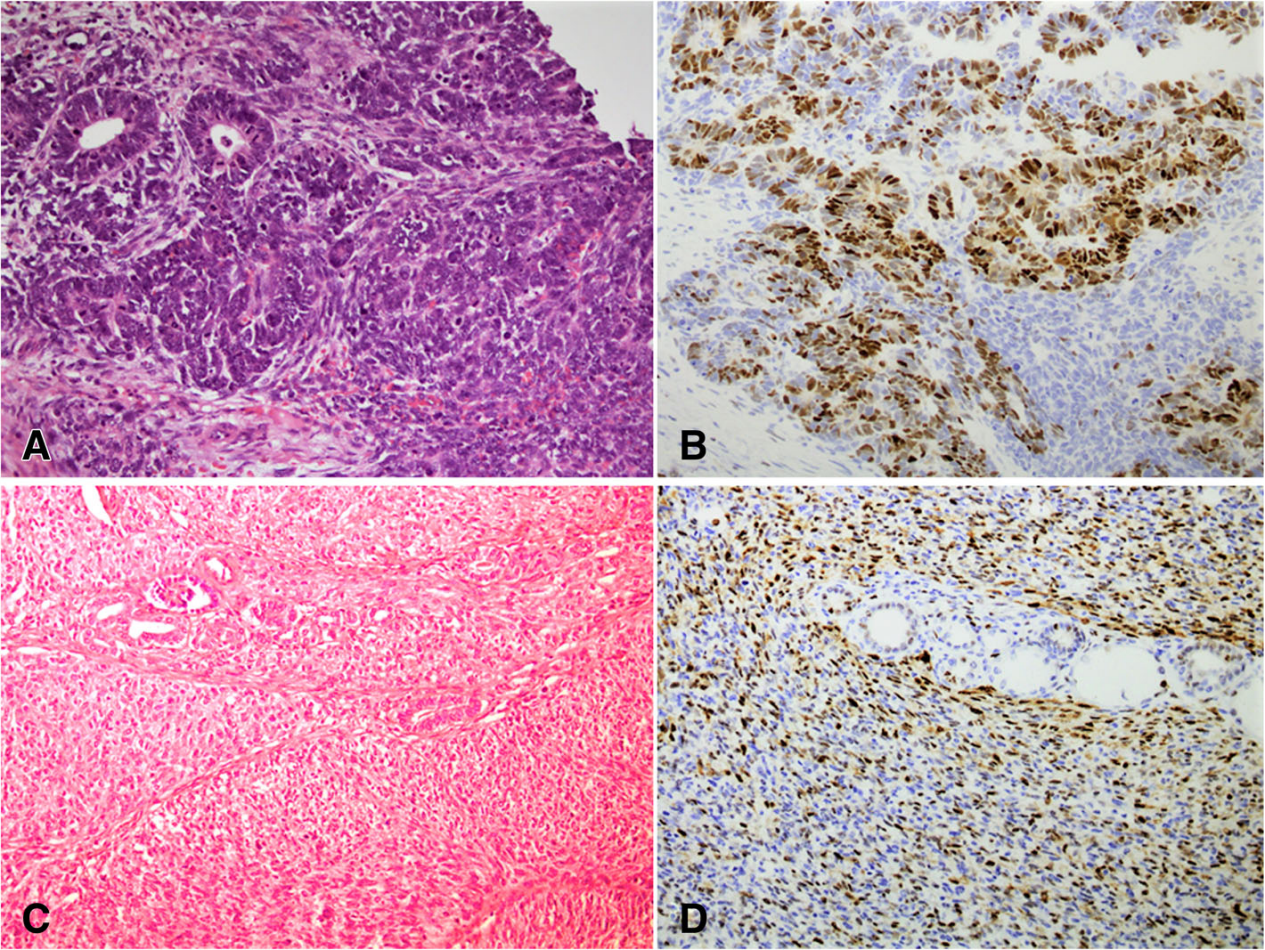
Classic Wilms tumor of kidney with epithelial and blastemal component (**a**). Cyclin D1 is strongly positive in the tubules while blastemal component shows rare positivity (**b**). Cellular congenital mesoblastic nephroma exhibiting trapped renal tubules (**c**), showing moderate to marked staining for Cyclin D1 (**d**)

Out of 9 cases of renal WT, 2 did not show any epithelial component and tumor was composed entirely of blastema and stromal components (biphasic). In these 2 cases, Cyclin D1 was negative in both components in 1 case, and showed weak positivity in blastemal component in the other case. The remaining 7 cases showed strong (3+) positivity in the epithelial component (tubules), while blastemal component was negative in all cases. Extent of staining ranged from 5 to 70%. Average extent of staining was 37.5% (Fig. 2 A,B). The details are shown in Table 2.

**Table 2:** Clinical details and results of immunohistochemical staining with Cyclin D1 and other IHC markers in Wilms tumor (*n* = 9)

Age (years)	Sex	Side	Size (cm)	Wilms tumor component	Cyclin D1 Intensity	Cyclin D1 Proportion (%)	Desmin	CKAE1/AE3	WT1	CD99
3	F	NK	NK	Blastema & stroma only; no epithelium	-ve	-ve	Focal +ve	NP	+ve	NP
2.6	F	L	17.5	Tubules	3 +ve	70	NP	NP	NP	NP
				Blastema	-ve					
3	F	R	8	Tubules	3 +ve	50	NP	NP	NP	NP
				Blastema	-ve					
6	F	R	10	Tubules	3 +ve	5	NP	NP	NP	NP
				Blastema	-ve					
5	F	L	15	Tubules	-ve	-ve	Patchy+	-ve	Patchy +ve	-ve
				Blastema	-ve					
4.5	F	L	7.5	Tubules	3 +ve	20	NP	NP	+ve	NP
				Blastema	-ve					
4	M	-	14	Tubules	3 +ve	40	NP	NP	NP	NP
				Blastema	-ve					
7	M	L	12	Tubules	3 +ve	45	+ve	+ve	+ve	NP
				Blastema	-ve					
6	M	R	9	Tubules	3 +ve	40	-ve	NP	+ve	NP
				Blastema	-ve					

*IHC* immunohistochemical, *M* Male, *F* female, *R* Right, *L* Left, *NP* Not performed, *NK* Not known

Ages of the 4 patients with congenital mesoblastic nephroma ranged from 15 days to 2 months. Out of 4 patients, 3 (75%) were males while 1 (25%) was female. Tumor was located in right kidney in 3 cases (75%) and left kidney in 1 case (25%). Tumor size ranged from 7 to 9 cm with mean size of 8 cm in largest dimension.

Out of 4 cases of congenital mesoblastic nephroma, 3 were cellular type and 1 was of mixed type and all demonstrated reactivity for Cyclin D1. Intensity of staining was 3+ in all 4 cases. Extent of staining ranged from 10 to 50% and average extent of Cyclin D1 staining was 30% (Fig. 2C, D). Anti-smooth muscle actin was performed in in 3 cases and was positive in all 3 cases.

Patients with renal RT ranged from 1 year to 14 years in age with mean and median age of 4 and 1.5 years respectively. Of the 4 patients, 3 (75%) were males and 1 (25%) was female. Tumor was located in left kidney in 2 cases (66.7%) and in right kidney in 1 case (33.3%). Tumor laterality was not known in 3 cases. Tumor size ranged from 7 cm to 9 cm in maximum dimension. Mean tumor size was 7.5 cm.

Out of 4 cases of renal RT, 3 (75%) demonstrated reactivity for Cyclin D1, intensity of staining was 2+ in all 3 cases while extent of staining ranged from 10 to 30%. Average extent of staining was 20%. Vimentin was positive in all 4 cases. EMA was performed in 3 cases and was positive in all 3. All 4 cases showed loss of INI 1.

The ages of 4 patients with renal Ewing sarcoma ranged from 2 to 36 years with mean and median age of 20 and 35 years respectively. Of the 4 patients, 2(50%) were males and 2 (50%) were females. Of the 4 cases, 3 were located in the right kidney (75%) while 1 (25%) was located in the left kidney (25%). Tumor size ranged from 2.5 to 17 cm in largest dimension. Mean tumor size was 11.7 cm.

**Fig. 3:**
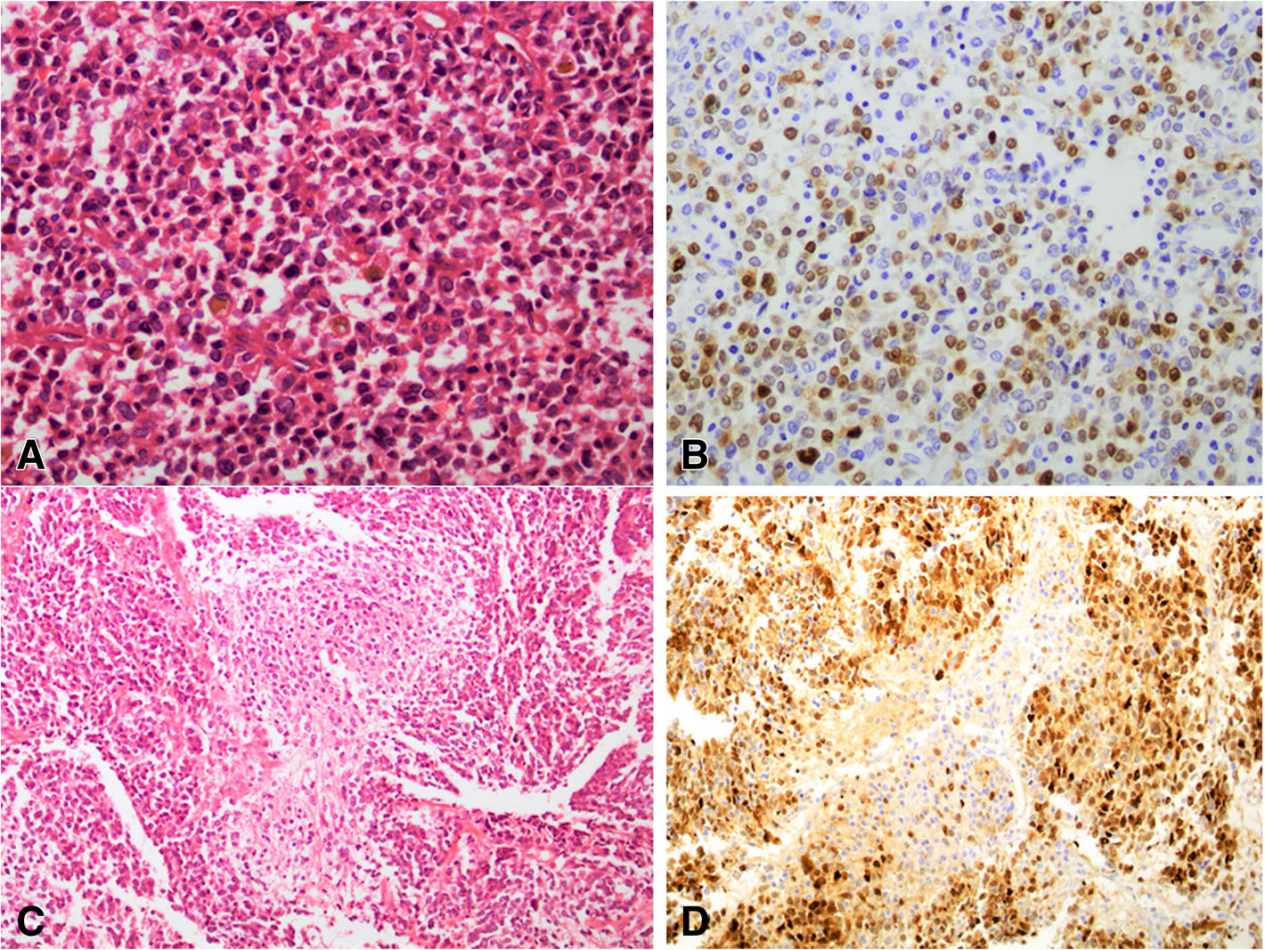
Renal Ewing sarcoma exhibiting (**a**) mild to moderate staining of Cyclin D1 (**b**). Neuroblastoma with central neuropil (**c**), showing diffuse strong Cyclin D1 positivity (**d**)

Out of the 4 cases of renal Ewing sarcoma, 3 (75%) demonstrated staining for Cyclin D1. Of these 3 cases, intensity of staining was 3+ in 2 cases (66.7%) and 2+ in 1 case (33.3%). Extent of staining ranged from 10 to 30%. Average extent of Cyclin D1 staining was 16.7% (Fig. 3A,B). CD99 was positive in all 4 cases.

We also included 8 cases of neurobastomas in our study. These 8 patients ranged from 0.9 to 25 years in age. Mean and median ages were 9 and 6 years respectively. Of the 8 patients, 5 (62.5%) were females while 3 (37.5%) were males.

Out of 8 cases of neuroblastoma in our series, 8(88.9%) demonstrated intense 3+ reactivity for Cyclin D1. Extent of Cyclin D1 staining ranged from 5 to 70%. Average extent of staining was 37.5% (Fig. 3 C, D). The details of Cyclin D1 staining are shown in Table 3.

**Table 3:** Clinical details and results of immunohistochemical staining with Cyclin D1 and other IHC markers in neuroblastoma (*n* = 8)

Age (years)	Sex	Site	Cyclin D1 Intensity	Cyclin D1 proportion	Synaptophysin	Neurofilament	CD56	Desmin	S100	CKAE1/AE3	Vimentin	WT1	CD99
25	F	Pelvis	3	40	+ve	+ve	+ve	NP	-ve	-ve	NP	NP	NP
10	F	Left kidney	3	90	+ve	+ve	+ve	-ve	+ve	-ve	Patchy +ve	Patchy +ve	NP
1	F	Pelvis	3	5	+ve	+ve	+ve	-ve	NP	NP	Focal +ve	-ve	NP
.5	M	Kidney	3	80	+ve	+ve	+ve	NP	NP	NP	NP	NP	NP
6	M	Sacroccocyx	3	70	+ve	-ve	+ve	-ve	NP	-ve	NP	NP	-ve
10	M	Inguinal LN	3	90	+ve	NP	+ve	NP	NP	NP	NP	NP	-ve
8	F	Adrenal	3	90	+ve	NP	NP	-ve	NP	NP	NP	NP	-ve
0.9	F	Presacral	3	90	+ve	+ve	+ve	-ve	NP	NP	NP	NP	NP

NP: Not performed; LN: Lymph node; M:Male;F:Female

## Discussion

All 19 cases of CCSK in our study demonstrated positivity for Cyclin D1 which was very intense (3+) in over 68% of cases and moderately intense (2+) in 21%. Thus over 89% cases showed moderate to marked intensity. Average extent of staining was almost 55%. These findings were indicative of diffuse and strong nuclear positivity for Cyclin D1 in the large majority of CCSK and were similar to the findings in other recent studies by Aw et al. (3) and Mirkovic et al. [5] which also showed moderate to severely intense diffuse positivity for Cyclin D1 in 7 out of 8 cases and all 14 cases respectively. These results demonstrate that Cyclin D1 is a very sensitive marker for CCSK.

All 4 cases of congenital mesoblastic nephroma (CMN) in our study showed markedly intense (3+) focal to diffuse staining for Cyclin D1. All 11 cases of CMN in Mirkovic et al’s study showed focal (4 cases) to diffuse (17 cases) moderate to severely intense Cyclin D1 staining [5]. This shows that Cyclin D1 is not useful in distinguishing between CCSK and CMN. CMN has distinct morphologic features with uniform fascicles of spindle cells that help in differentiating it from CCSK [3]. Molecular methods can also help (the cellular form of CMN can mimic CCSK morphologically but its distinct translocation can be identified by PCR) [20].

Similarly, 7 out of 9 cases of WT in our study demonstrated intense 3+ staining for Cyclin D1 in their epithelial component with average extent of staining of almost 40%. All 4 cases of NB in Mirkovic et al.’s study also demonstrated diffuse moderate to strong nuclear staining for Cyclin D1 [5]. Thus, Cyclin D1 is verye useful in distinguishing between CCSK and WT in cases in which blastema predominates (blastema-rich WT), as Cyclin D1 will highlight the epithelial component. Since the prognosis and therapy of CCSK and WT are different, it is important to distinguish between the two and incidentally the most difficult morphologic distinction in the differential diagnosis of CCSK is blastema-rich WT [5].

Of the 4 cases of renal Ewing sarcoma in our series, 3 (75%) demonstrated focal to diffuse staining for Cyclin D1 which was very intense in 2 cases. All 5 cases of renal Ewing sarcoma in the study by Mirkovic et al. [5] showed focal (1 case) to diffuse (4 cases) staining of variable intensity for Cyclin D1. Thus Cyclin D1 cannot reliably distinguish between CCSK and Ewing sarcoma. The latter however has a distinct morphologic appearance and a specific translocation i.e. t(11,22) seen in about 85% of cases which can help in distinguishing it from CCSK [21].

Of the 4 cases of renal RT in our study, 3 (75%) showed moderately intense staining for Cyclin D1 which ranged in extent from 10 to 50% with average extent of staining of 30%. This finding is in contrast to the results in the study by Mirkovic et al. [5]. Of the 4 cases of RT in their study, 2 were negative, while the other 2 showed focal sparse nuclear staining for Cyclin D1. Both Mirkovic et al. [5] and Aw et al. [3] believe that Cyclin D1 is useful in differentiating CCSK from renal RT but our findings indicate otherwise. However, number of cases of renal RT in our study was very small to derive any definite conclusions. In equivocal cases, the loss of nuclear positivity for INI 1 immunohistochemical marker in RT can be helpful in differentiating it from CCSK [22]. All 4 cases of RT in our study showed loss of INI 1.

All 8 cases of neuroblastoma in our study demonstrated intense 3+ staining for Cyclin D1 which ranged in extent from as low as 5% to as high as 90%. In fact, 6 out of 8 cases (75%) showed diffuse 3+ positivity which ranged in extent between 70 and 90%. This finding is similar to the findings by Mirkovic et al. [5] and Aw et al. [3] and confirms their results that Cyclin D1 immunohistochemistry cannot be used to reliably distinguish CCSK from Neuroblastoma.

As discussed by both Mirkovic et al. [5] and Aw et al. [3], Cyclin D1 is negative or shows only rare or sparse nuclear positivity in the diffuse blastematous and stromal components of WT while epithelial component may show strong and diffuse nuclear positivity or may even be negative. In our study, 7 out of 9 cases of WT demonstrated intense (3+) Cyclin D1 positivity in the epithelial component and extent of staining averaged around 40%. Cyclin D1 is thus helpful in differentiating CCSK from blastema-dominant WT which mimics CCSK morphologically. Although Cyclin D1 is positive in both CCSK and the epithelial component of WT, the two can usually be differentiated from each other by their morphological features which are distinct. Thus our findings, in relation to Cyclin D1 positivity in WT were similar.

## Conclusions

Cyclin D1 is a sensitive but not specific immunohistochemical marker for CCSK and many other pediatric renal malignant neoplasms as well as for neuroblastoma. Hence, careful examination of histological features is important in reaching an accurate diagnosis in CCSKs. Cyclin D1 however is very helpful in distinguishing between CCSK and blastema-rich WT which is arguably the most difficult morphologic distinction in the differential diagnosis of CCSK. Our results were similar to two other recently published studies [3, 5]. However, the small number of cases is a limitation of our study. Larger studies are recommended in order to verify and validate these results.

## Abbreviations

CCSK: Clear cell sarcoma of kidney
CMN: Congenital mesoblastic nephroma
NB: Nephroblastoma
NWTS: National Wilms Tumor Study
RT: Rhabdoid tumor
WT1: Wilms Tumor 1
